# Synthesis and Applications of Molecularly Imprinted Polymers Modified TiO_2_ Nanomaterials: A Review

**DOI:** 10.3390/polym10111248

**Published:** 2018-11-11

**Authors:** Lingna Sun, Jie Guan, Qin Xu, Xiaoyu Yang, Juan Wang, Xiaoya Hu

**Affiliations:** 1 College of Chemistry and Chemical Engineering, Yangzhou University, Yangzhou 225002, China; sunlna9610@163.com (L.S.); 18852726503@163.com (J.G.); yangxiaoy77@163.com (X.Y.); 15705276738@163.com (J.W.); 2 Guangling College, Yangzhou University, Yangzhou 225002, China

**Keywords:** review, titanium dioxide, nanocomposites, molecular imprinting, synthesis, application

## Abstract

Titanium dioxide (TiO_2_) nanomaterials have caused a widespread concern in the past several decades for their bulk characteristics and potential applications in many different areas. Lately, the combination between molecularly imprinted polymers (MIPs) and TiO_2_ nanomaterials have been proven to improve the relative adsorption capacity, selectivity and accelerate the rate of mass transfer of analyte which is not possible using TiO_2_ alone. Considering the unique performance of the MIPs modified TiO_2_ nanomaterials, this review intends to give an overview of the recent progresses in the development of MIPs modified TiO_2_ nanomaterials, the potential applications of their tailor-made characteristics. The limitations and challenges in this practically promising nanomaterials have also been raised and summarized. By means of the points raised in this article, we would like to provide some assistance for further development of preparation methodologies and the expansion of some potential applications in the field of MIPs modified TiO_2_ nanomaterials.

## 1. Introduction

Titanium dioxide (TiO_2_) is a photosensitive substances which can produce conduction band electrons, valence holes, superoxide radicals, hydroxyl radicals and other active species under the excitation of light and then lead to a variety of organic compounds degradation [1], killing most of the microbes [2]. As a green photocatalyst, TiO_2_ nanomaterials have attracted a lot of attention in the field of photochemistry. However, their applications are limited by their poor selectivity because of the strong non-selective oxidizability of holes and hydroxyl radicals generated by TiO_2_ excited by ultraviolet light. When the target analytes were in a mixture or their concentrations are low, the application efficiency of TiO_2_ nanocomposites would be severally hampered. Several procedures such as the surface’s electric charge control, double-region-structured photo-catalysts design procedure et al. have been developed to improve the selectivity of TiO_2_ nanocomposites but these nanocomposites could only remove organics with charges but not the uncharged ones [3]. Therefore, using suitable organic or inorganic materials with special selectivity to modify TiO_2_ nanocomposites is imperative to guarantee their functionality for specific recognition.

Polymers are a kind of modifiers that have been widely implemented for the modification of TiO_2_. The polymer modification on TiO_2_ could alter their hydrophobic/hydrophilic character, improve their dispersion in various media and introduce new functional groups for the reaction with organic molecules [4]. But polymers modified on TiO_2_ prepared by direct mixing, sol-gel processing and in situ grafting polymerization process lack selectivity. 

Molecular imprinting technology (MIT) offers the opportunity to endow TiO_2_ based materials good selectivity because they can composite tailor-made receptors which can selectively recognize and bind target molecules with high affinity. This technology usually refers to a progress that the template molecules interact with the selected functional monomer to form main host-guest complexes and then a certain amount of cross-linking agent and initiator were added together into the complexes to obtain macromolecule polymers. Upon removing the template molecule, a recognition cavity which is complementary with the template molecule in shape, size and chemical functionality is formed in the highly cross-linked polymer matrix, so that the obtained MIPs have higher recognition ability and can selectively separate the specific template molecules [5]. This technique can be described as a ‘manual locking’ technique for identifying ‘molecular bonds’. MIPs have three distinct characteristics [6]: (1) Structure-activity predictability. Different MIPs can be prepared according to the working purposes to meet different needs. (2) Specific recognition. MIPs prepared according to the structure of the template molecule have specific recognition of the template molecule. (3) Wide practicability. The performance of MIPs can be compared with natural molecular recognition systems such as antigens and antibodies, enzymes and substrates, receptors and hormones [7]. MIPs are synthesized by chemical methods and have the advantages of high stability and long-term reusability. What’s more, MIPs can make up for the shortcomings of bimolecular which cannot be used in extreme environments [8] (such as strong acids, strong bases and organic solutions). 

MIPs have been widely used in solid phase extraction, sensors construction, membranes, catalysts and drug delivery. Several reviews related to the development and application of MIPs have been reported [9,10,11,12]. Chen [13] published a review about imprinting methods, challenges and effective strategies for MITs and some significant applications of them. Wackerlig [14] reviewed MIPs’ application about analytical separation, artificial antibodies and in vivo applications. Gui [10] reported a review on molecularly imprinted sensors. Since MIPs have so many advantages and such widespread and multidisciplinary applications, novel techniques of MIP deposition, surface development of MIP films or introduction of unique properties are demanded in terms of selective and sensitive MIP layer fabrications. Special attentions have been paid to the synthesis of MIPs based nanomaterials by using nanoparticles as supports. The nanomaterials combine properties of both nanoparticles and MIPs. That is, the composites have the good selectivity of MIP layers and good optical, electrochemical or magnetic properties of the nanoparticles. This form of MIPs is very promising for the practical applications and fundamental research. Multiwalled carbon nanotubes [15], metal-organic framework [16], ZnO [17,18], magnetic nanoparticles [19], silica particles [20] et al. have been used as the support for molecular imprinting. Niu [9] had reported the recent trends in core-shell nanoparticles coated with MIPs (core-shell MIPs). Yáñez-Sedeño [11] published a review which focused on the recent progress of magnetic-molecular imprinted polymers (MMIPs). Dai [12] reviewed on the recent developments of molecular imprinting techniques and applications based on the surface of carbon nanotubes (CNTs). A multitude of materials [21] (i.e., not limited to TiO_2_ composites) for molecular imprinting and/or the application of MIPs in different areas have been reported [14,22]. The study of MIPs modified TiO_2_ nanocomposites is one research focus and many articles in this area are updated each year. Table 1 listed the advantages and disadvantages of MIPs modified TiO_2_ nanomaterials. Theses nanomaterials have been widely used in the field of sensor construction, separation process, pollutant removal and drug development. Nevertheless, a timely and overall review of the current development of MIPs modified TiO_2_ nanomaterials is lacking. This review intends to introduce the MIPs modified TiO_2_ nanomaterials during the past 20 years from the aspects of synthesis, related applications and prospects, furthermore, the challenges presently encountered and some feasible resolutions. By means of the points raised in this article, we would like to provide the understanding of the importance of the MIPs modified TiO_2_ nanomaterials, some assistance for further development of preparation techniques and the expansion of their potential applications.

## 2. General Method for the Preparation of MIPs Modified TiO_2_ Nanomaterials

Different approaches have been performed for the integration of TiO_2_-based nanomaterials into the MIPs matrix in the last decades via varieties of methods. These methods are generally classified as surface imprinting, precipitation polymerization and in situ polymerization. The following discussions summary each of the three general common methods and stress researches interest from the literature.

### 2.1. Surface Molecular Imprinting Technique

**Figure 1:**
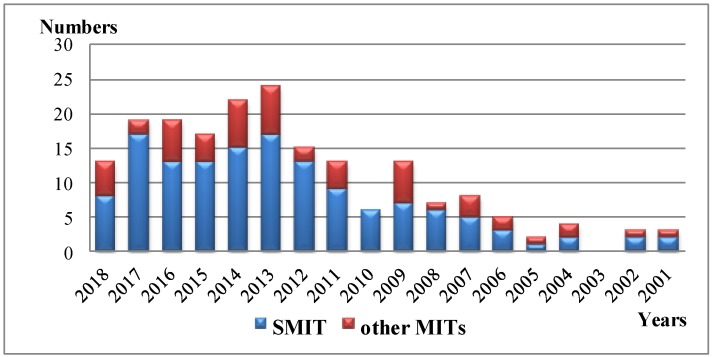
The annual numbers of journal publications on SMIT and other MITs from 2001 to 2018 (originated from Web of Science^TM^) via searching publications including “TiO_2_ molecular imprint” and “TiO_2_ surface molecular imprint” (SMIT) in the topic.

Surface molecular imprinting technique (SMIT) establishes a molecular recognition system with fitting binding sites for specific target molecules on the surface of a solid support or matrix material. Figure 1 displays the synthetic methods of MIPs modified TiO_2_ nanomaterials in the last decades. The introduction of the surface imprinted technique allows MIP particles to present highly uniform size and shape and the presence of recognition sites on the surface the substrate. As a consequence, the migration and combination of the reactive substances are fast, which is favorable for accelerating the reaction and reducing the embedding phenomenon. It also makes it easier to separate elution blotting molecules while increasing application efficiency.

As SMIT can not only imprint general small molecules but also apply to biological macromolecules such as proteins [26,27,28,29,30,31,32], this technology is gaining more and more attention in the field of molecular imprinting. Surface molecularly imprinted polymerization techniques include graft copolymerization, sacrificial carrier method, sol-gel method, sol-hydrothermal polymerization and so on.

#### 2.1.1. Graft Copolymerization

Surface grafting copolymerization process is realized through the covalent reaction between different functional groups on the surface of the TiO_2_ based nanomaterials and the grafting polymer brushes. Thin MIPs layer could be obtained through the grafting approach. The obtained polymer recognition site is located on the surface of the carrier, which facilitates the target to quickly approach the recognition site and has a higher binding rate, thereby reducing the nonspecific adsorption. Imprinting molecules on the surface of TiO_2_ nanomaterials can give the material a richer function and make it more conducive to applications in various fields.

**Figure 2:**
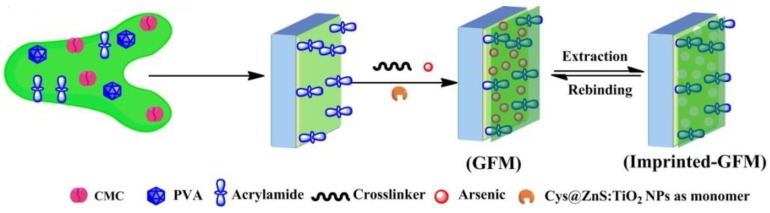
Graphical representation for the fabrication of imprinted membrane synthesized via ‘grafting-from’ (GFM) approaches. Reprinted with permission from ref [34]. Copyright 2016 American Chemical Society.

Yao et al. [33] synthesized TiO_2_ hybrid molecular imprinted polymer by using bensulfuron-methyl (BSM) as the template molecule, methacrylic acid (MAA) as the functional monomer, and silane coupling agent 3-(trimethoxysilyl) propylmethacrylate (KH570) as organic–inorganic connective bridge. The obtained MIPs have stable chemical property, high mechanical strength, large specific surface area and adsorption capacity, good selectivity and easy desorption. Roy et al. [34] fabricated a membrane which exhibited high adsorption capacity with outstanding specific selectivity towards As (III) and As (V). They used a cysteine (Cys) derivative modified TiO_2_ doped ZnS nanoparticle (Cys@ZnS:TiO_2_ NPs) as the monomer. The selective he membrane was prepared by the combination of ‘grafting-from’ and MIT (Figure 2). In this procedure, acrylamide (AA) was firstly mixed with membrane precursors (carboxymethyl cellulose, CMC and polyvinyl alcohol, PVA) to form a base membrane. Then, a pre-polymer mixture which contained template (As (III) or (V)), functional monomers Cys@ZnS:TiO_2_NPs), cross-linker (N-N′-methylene bisacrylamide) and an initiator (APS) was added to the base membrane to generate the imprinted membrane. The adsorption capacity of this membrane towards As (III) and As (V) is 151.0 and 130.0 mg/g, respectively. Yang et al. [35] prepared a chemiluminescent sensor based on nitrobenzoxadiazole(NBD)-grafted anatase nanoparticles for detecting phenoxyacetic acid compounds sensitively and selectively, such as the herbicide 2,4-dichlorophenoxyacetic acid. This chemiluminescence sensor was constituted of anatase nanoparticles grafted with the NBD fluorophore and bis(2,4,6-trichlorophenyl)oxalate (TCPO. Firstly, anatase TiO_2_ nanoparticles were functionalized with a hybrid monolayer of the NBD fluorophore and amino groups, then it was covalently linked with 3-aminopropyltriethoxysilane (APTS) by a nuclephile reaction. The sensor had a very low detection limit of 0.33 nM and can promote the development of sensors based chemiluminescent nanomaterials.

#### 2.1.2. Sacrificial Carrier Method

The sacrificial carrier method fixes the imprinted molecule on the surface of the carrier in a solvent and removes the imprinted molecule and dissolves the carrier when the polymerization reaction is finished. During the procedure, the template molecules are firstly immobilized on the surface of the solid support by chemical bonding and then the support is placed in the monomer solution for polymerization. After the polymerization reaction is completed, the carrier is chemically dissolved and the template molecule is eluted to obtain a molecularly imprinted polymer having a binding site on the surface. 

**Figure 3:**
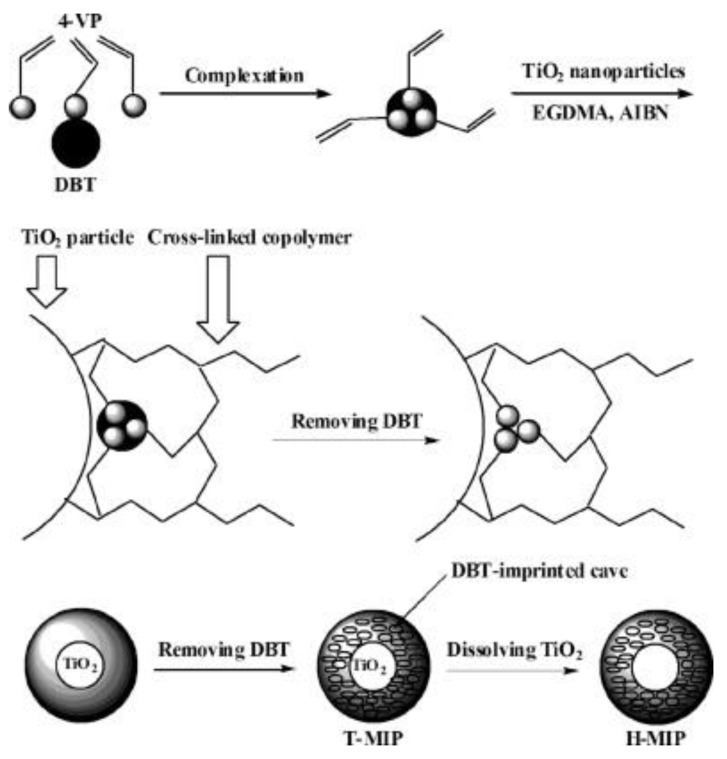
Schematic representation of the route for the synthesis of H-MIPs. Reprinted with permission from ref [36]. Copyright 2011 American Chemical Society.

Xu et al. [36] prepared a novel MIPs based on surface imprinting technique with nano-TiO_2_ as a sacrificial support matrix, dibenzothiophene (DBT) as the imprinted molecule, 4-vinylpridine (4-VP) as a functional monomer and ethylene glycol dimethacrylate (EGDMA) as a cross-linker. As illustrated in Figure 3, they composited the imprinted mixture on the surface of nano-TiO_2_ during the polymerization and then, dissolved and removed nano-TiO_2_ to obtain uniformly hollow particles which consist of the imprinted polymer (H-MIPs). Li et al. [37] prepared hollow chlorogenic acid imprinted polymer by using nano-TiO_2_ as a sacrificial support matrix, 4-VP or MAA as a functional monomer and EGDMA as cross-linker.

#### 2.1.3. Sol-Gel Polymerization

Sol-gel process usually starts with dissolving metal or semimetal alkoxide in alcoholic or other organic solvents to form a solution and then adds a little amount of water to initiate the hydrolysis and condensation reaction. With the process of the reaction, the viscosity of the matrix increases which means the transformation of sol into the rigid, porous and network-like gel. After aging, the final product was formed.

Sol-gel technique was also used to prepare molecularly imprinted membranes by dip-coating and spin-coating on a substrate. This method has the advantages of low synthesis temperature [38] high purity, uniform film formation, simple process and easy doping. A large number of articles have summarized the sol-gel process and their applications as well as their physical and chemical properties [39]. Sol-gel preparation of mlecularly imprinted materials always involves three steps. The first step is the selection of the template. The second step is the incorporation of the template into the polymer network and the last step was the removal of the template. 

**Figure 4:**
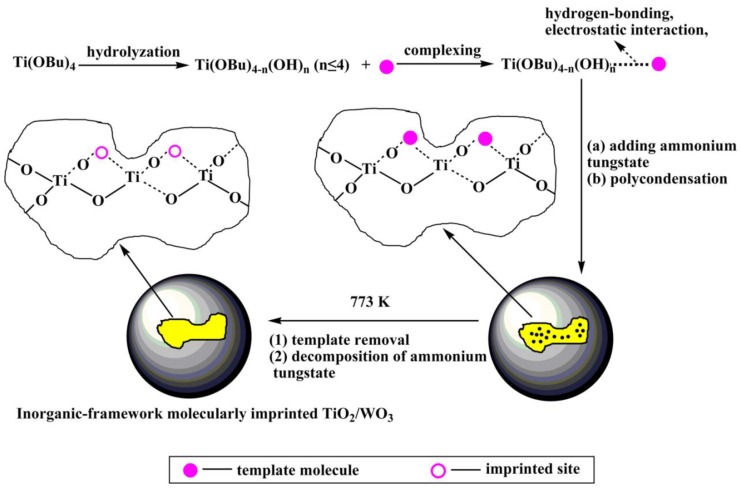
Route for preparation of inorganic–framework molecularly imprinted TiO_2_/WO_3_ nanocomposite. Reprinted with permission from ref [42]. Copyright 2013 American Chemical Society.

Takahara et al. [40] deposited *β*-cyclodextrin/bisphenol A (*β*-CD/BPA) complex and Ti(O-Bu-n)_4_ alternately on a quartz crystal microbalance (QCM) to prepared the bisphenol A imprinted TiO_2_ film by sol-gel method. The imprinted TiO_2_/(*β*-CD/BPA, 2:1) film showed about 7-fold higher selectivity than the non-imprinted TiO_2_/beta-CD film and a sensitivity lower than 50 ppb to BPA. Wei et al. [41] utilized the inorganic Fe_3_O_4_@SiO_2_ composite as the imprinted matrix, 4-nitrophenol as a template molecule, and Ti(oBu)_4_ as a cross-linking agent to prepare the final core-shell molecularly imprinted TiO_2_/WO_3_-coated magnetic nanocomposite. The degradation rate of this composite for 4-nitrophenol was 2.5 times that of the non-imprinted nanocomposite. Luo et al. [42] prepared inorganic-framework molecularly imprinted TiO_2_/WO_3_ nanocomposites with molecular recognitive photocatalytic activity using the sol-gel method by using 2-nitrophenol and 4-nitrophenol as template molecules and tetrabutylorthotitanate as a titanium source and the precursor to functional monomer (Figure 4). The molecularly imprinted TiO_2_/WO_3_ exhibits higher stability and selective than non-imprinted TiO_2_/WO_3_. Cai et al. [43] used metallothionein (MT) as template and TiO_2_ sol as imprinting matrix to synthesize MT blotted TiO_2_ films by surface sol-gel method. According to Li et al. [44], SMIT combined with sol-gel process was applied to synthesis a new Pb(II)-imprinted polymer with nano-TiO_2_ as a solid substrate, glycidoxy propyltrimethoxysilane (GPTMS) as both a crosslink and a silane coupling agent. Song et al. [45] prepared the new molecularly imprinted inorganic-framework Fe–TiO_2_ composites (MIPs/Fe–TiO_2_) based on the sol-gel method with acid orange II (AOII) as the template molecule and TiO_2_ as the matrix material. Liu [46] used nnthracene-9-carboxylic acid as the imprinting molecular to construct a novel molecularly imprinted TiO_2_ thin film modified TiO_2_ nanotube array photocatalyst by the sol-gel method. Compared with unmodified TiO_2_ nanotube and non-imprinted TiO_2_ film modified TiO_2_ nanotube, the MIPs modified TiO_2_ nanotube has higher adsorption capacity for target pollutants and enhanced photocatalytic activity in the photodegradation of pollutants. 

#### 2.1.4. Sol-Hydrothermal Polymerization

Sol-hydrothermal method is a combination of the sol-gel and hydrothermal synthesis process. This method can not only reduce the reaction temperature but also avoid high-temperature calcinations so that cannot influence the grain growth. What’s more, it promotes full contact between the reactants, so that the reaction can be fully carried out. 

According to Deng et al. [47], they used 4-nitrophenol (4-NP) as template through sol-hydrothermal method and then prepared inorganic-framework molecularly imprinted TiO_2_/SiO_2_ nanocomposite (MIPs-TiO_2_/SiO_2_) successfully. Later, Deng et al. [48] prepared inorganic-framework molecularly imprinted TiO_2_ nanoparticles by sol-hydrothermal method using tetrabutylorthotitanate as titanium source as well as precursor of functional monomer and 4-NP as template. The obtained MIPs-TiO_2_ is highly reusable for its stable inorganic framework and the facilely released active sites during regeneration process.

### 2.2. Precipitation Polymerization

Precipitation polymerization, also known as heterogeneous solution polymerization, means that the functional monomers, cross-linking agents and initiators used in the polymerization are dissolved in a dispersant to form a homogeneous mixed solution. The resulting polymer is insoluble in the reaction medium and precipitates. Precipitation polymerization method can be divided into two stages. Firstly, oligomers formed in the two-phase interface reach a certain concentration by crosslinking nucleation and then gather together into polymer particles. Secondly, these particles do not overlap or coalesce but can capture oligomers and functions monomers in the diluted reaction system to grow up individually [49] and eventually form uniform and highly crosslinked polymer microspheres [50,51]. The final morphology of the obtained polymers is directly influenced by the template [52] and functional monomer [53] which are used in polymerization. This method does not need to add any surfactants and stabilizers in the polymerization process. Therefore, the surface of the prepared polymer microspheres is clean, which can effectively avoid the non-selective adsorption of the imprinted molecules by surfactants and stabilizers [54]. This review describes two precipitation polymerization producers: the liquid deposition and precipitation methods.

#### 2.2.1. Liquid Deposition Method (LPD)

Liquid deposition is a process of spontaneous deposition of crystals from a supersaturated solution or forming a thin film on the substrate by adding reactants which can react with the raw materials. Currently, the liquid deposition method mainly uses metal fluoride as a reaction precursor. The metal fluoro-complex ion ([MF_n_]^m-n^) undergoes a ligand displacement reaction with the fluoride ion-depleting agent in the solution to promote the hydrolysis equilibrium of the metal fluoride [55], thereby depositing the metal oxide to form a thin film. The method deposits a metal oxide film on the surface of the substrate by adding boric acid (H_3_BO_3_), water or aluminum metal to [MF_n_]^m-n^ solution. Boric acid not only be used as a fluoride ion-consuming agent but can react with hydrogen fluoride to generate water to further promote the reaction [56].

**Figure 5:**
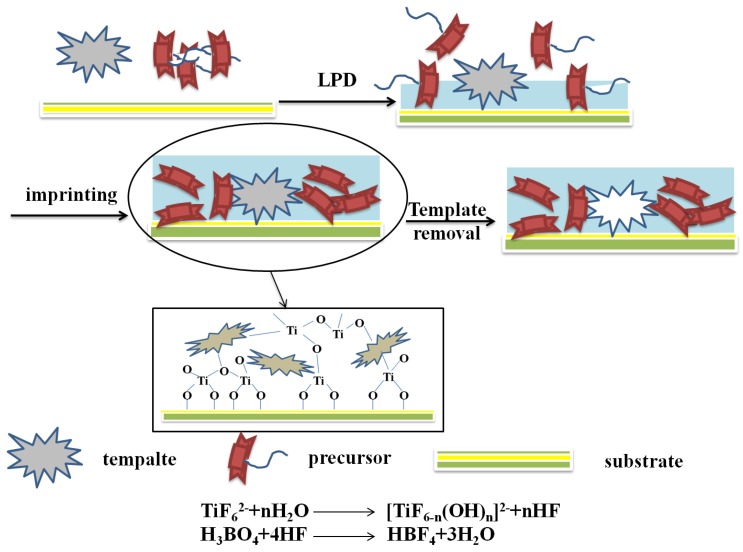
Scheme and process for preparation of MIPs modified TiO_2_ nanomaterials by the LPD method.

Figure 5 illustrated the MIPs modified TiO_2_ nanomaterials via the LPD procedure. When liquid phase deposition method was used to prepare the molecularly imprinted TiO_2_ thin films, the template molecules are usually added into (NH_4_)_2_TiF_6_ and H_3_BO_3_ precursor solution to obtain TiO_2_ thin films containing template molecules. The template molecules are then removed by solution washing or UV radiation. This method is a typically homogeneous mixing system based on a liquid phase. Therefore, multi-component oxide films with uniformly distributed imprinting sites can be synthesized easily. In addition, this method has the advantages of low processing temperature, simple equipment, high selectivity, uniform film, high film quality, low cost [57], and so on.

Wang et al. [58] prepared the imprinted TiO_2_ films by using acetaminophen as a template molecule and p-tert-butyl calixarene as a functional monomer in the presence of (NH_4_)_2_TiF_6_ and H_3_BO_3_.The imprinted acetaminophen can be removed completely by washing with ethanol. Wang, H. et al. [59] synthesized tetracycline hydrochloride (TC) molecularly imprinted titania modified TiO_2_ nanotubes by added template molecule TC into (NH_4_)_2_TiF_6_ and H_3_BO_3_ precursor solution. The obtained MIPs can improve the molecular recognition ability of the photocatalyst toward template molecules. Feng et al. [60] used L-glutamic acid (GA) as template molecule and synthesized the GA-imprinted TiO_2_ films by liquid-phase deposition in the presence of (NH_4_)_2_TiF_6_ and H_3_BO_3_. Tatemichi et al. [61] added the template molecule pepsin to the (NH_4_)_2_TiF_6_ precursor solution to deposit the nano-TiO_2_ along with the complex of pepsin and polylysine onto the gold substrate. The place where the complex was retained in the template membrane after deposition and the pepsin was subsequently removed to prepare a molecularly imprinted nanoparticle coating containing pepsin holes. Xu et al. [62] synthesized the molecularly imprinted TiO_2_ hybridized magnetic Fe_3_O_4_ nanoparticles by using LPD method with estrone as a template molecule and then the target estrone can be removed with the irradiation of UV light. 

#### 2.2.2. Seed Precipitation Polymerization

**Figure 6:**
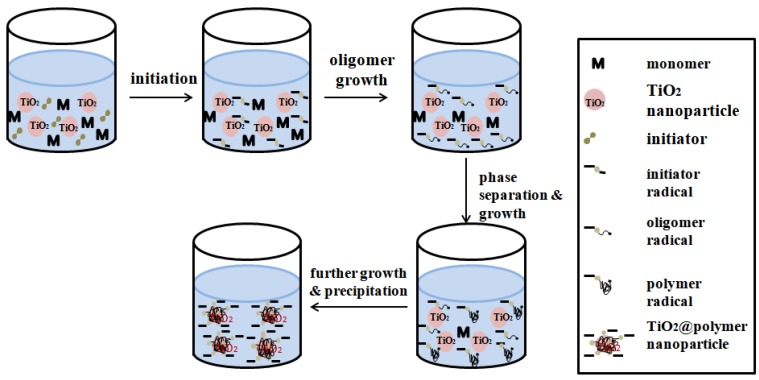
Schematic depiction of the seeded precipitation polymerization mechanism: (1) homogeneous solution of monomers, TiO_2_ seeds and initiator molecules; (2) Thermal decomposition of the initiator leads to initiator radicals; (3) oligomer radicals’ growth; (4) growth of polymer radicals and phase separation; (5) precipitation of oligomer/polymer radicals onto the surface of the TiO_2_ nanoparticle seeds. Adapted from ref [65].

Seed precipitation polymerization (Figure 6) is a typical multi-step swelling and polymerization method. In the initial stage of reaction, when some monomers reach the limit in the aqueous phase, basic particles are deposited. When these basic particles are very tiny, the seed particles will be adsorbed and form a shell layer on the seed surface, which will then become the polymerization reaction of MIPs place. Compared with the traditional molecular imprinting method, the polymer synthesized by ‘seed precipitation polymerization’ has high affinity and selectivity, more easily available sites and more uniform [63].

Huang et al. [64] used nano-TiO_2_ as the support matrix, 4-VP as the functional monomer, kaempferol as the template molecule, ethylene glycol dimethacrylate (EDMA) as the cross-linking agent to prepare kaempferol imprinted polymers and nano-TiO_2_-based MIPs were obtained at last after removing the template. The polymer particles made in this work appear as uniform microspheres with high selectivity and template recognition.

### 2.3. In Situ Polymerization

In-situ polymerization is the method of synthesizing a MIPs solid phase in a specific container (such as chromatographic column) by mixing solvent, template molecule, functional monomer, cross-linking agent and initiator in a certain ratio [66,67]. Because this polymerization method is completed in one step in the column without the need of grinding, sieving, sedimentation and other processing steps, the preparation process is straightforward and can be directly used for analysis with strong practicability.

At present, the method of directly polymerizing MIPs on the surface of nanomaterials by ultraviolet light irradiation is widely used for its advantages of easy reaction process, easy operation, less dosage of initiator or cross-linking agent and high conversion rate [68,69]. UV irradiation is used to initiate the polymerization reaction, which has two advantages compared to thermal initiation. First, at low temperatures, strong complexes can be formed between template molecules and functional monomers, so UV light has been proved to be beneficial for MIPs synthesis which can be applied at extremely low temperatures. Second, UV light can also be used as an effective approach for preparing a detection window and controlling the overall length [70].

In comparison to other methods, the in situ synthesis of TiO_2_-MIPs remains far less explored. Shen et al. [71] used excessive ortho-phenylenediamine (OPDA) as the monomer and the target compound (4-chlorophenol (4CP) or 2-chlorophenol (2CP)) as the template and then the MIPs layer was coated on the surface of TiO_2_ particles through in-situ polymerization under UV irradiation. The finally MIPs-coated TiO_2_ molecularly imprinted photocatalyst was obtained by removing the template molecules. 

## 3. Application of TiO_2_ and Their Composites Based Molecularly Imprinted Polymers

The combination of TiO_2_ nanomaterials with molecular imprinting technology can enhance its stability and photocatalytic activity, improve its selectivity and broaden their application scope. Based on the TiO_2_ nanomaterials and molecular imprinting properties, we mainly review the application of these materials in three aspects: selective photocatalysis, electrochemical and photoelectric sensing and other applications.

### 3.1. Application in Photocatalytic Degradation

In 1972, Japanese scholars Fujishima and Honda reported the results of research using hydrogen peroxide to decompose water with TiO_2_ under UV light irradiation [72], the application of semiconductor materials in photocatalytic degradation has been further studied [73]. TiO_2_ has been widely used in the photocatalytic degradation of environmental pollutants due to its suitable electronic band structure [74], biological and chemical inertness [75], strong oxidizing capability, light stability and non-toxicity, which make it to be one of the most promising photocatalyst one [76,77,78]. However, TiO_2_ shows poor photocatalytic selectivity because photocatalytic reactions based on TiO_2_ are accompanied by the formation of highly reactive ·OH radicals which are typically nonselective [79]. Many of the researches have examined that the combination of MIPs with TiO_2_ and its composite materials can improve the special recognition and selectivity of TiO_2_ photocatalytic degradation [42,80,81,82,83], which is of great significance for the photocatalytic degradation of industrial wastewater [3]. Table 2 listed the details of some of the MIPs modified TiO_2_ nanomaterials and their applications for photocatalytic degradation.

**Figure 7:**
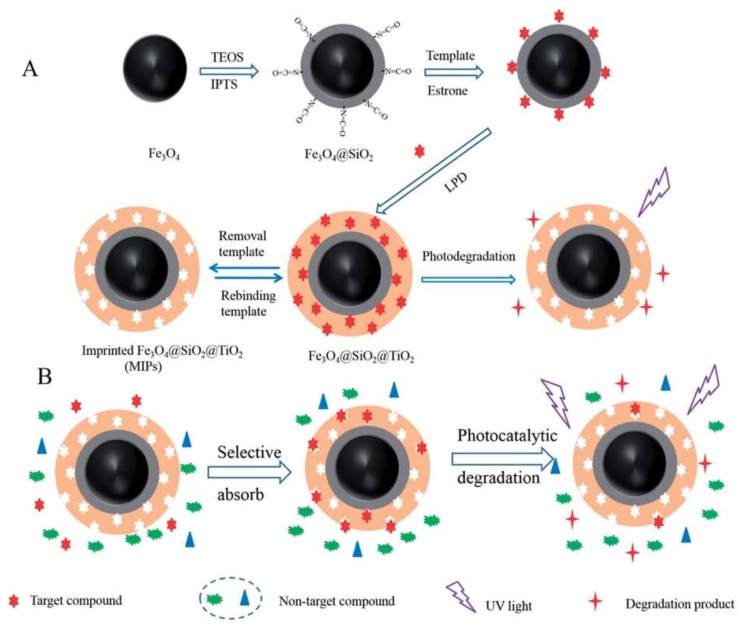
The Schematic illustrations for preparation estrone imprinted TiO_2_ films on the surface of magnetic Fe_3_O_4_ nanoparticles (**A**) and the possible mechanism of selective photocatalyst degradation of target template by imprinted Fe_3_O_4_@SiO_2_@TiO_2_ (**B**). Reprinted with permission from ref [62]. Copyright 2014 American Chemical Society.

Environmental Estrogens (EEs) endanger the body’s endocrine system and affect the growth and reproductive functions of humans and animals [94]. Removing estrogenic chemicals from wastewater is a matter of great concern. Xu et al. [62] used estrone as the template molecule to prepare molecularly imprinted TiO_2_ hybridized magnetic Fe_3_O_4_ nanoparticles by a LPD method. The estrone can be selectively degraded and removed under the irradiation of UV light (Figure 7). The obtained Fe_3_O_4_@SiO_2_@imprinted TiO_2_ demonstrated high adsorption and capacity selectivity, fast kinetics and excellent stability during long-time photocatalysis. The theoretical maximum adsorption amount of EESs on the Fe_3_O_4_@imprinted TiO_2_ was 2.62 mg/g. This material can provide a potential application prospect for photocatalytic degradation and removal of trace target organic pollutants in the presence of high-level pollutant. Zhang et al. [85] took precipitation polymerization to synthesize the imprinted polymer-modified TiO_2_ nanotubes (S-MIP-TiO_2_ NTs) by using 17*β*-estradiol as the template, MAA as the functional monomer, trimethylolpropanetrimethacrylate as the crosslinking agent, 4,4′-azobis (4-cyanovaleric acid) as the initiator. The experimental results showed that the adsorption range of S-MIP-TiO_2_ NTs was from 10 ng/L to 1000 mg/L and the apparent first-order rate constant kinetics (k_app_) was 0.0732 min^−1^ for S-MIP-TiO_2_ NTs. It demonstrated higher adsorption strength and selectivity for photocatalytic degradation at low concentrations of 17*β*-estradiol compared with pure TiO_2_. In addition, S-MIP-TiO_2_ NTs photocatalyst has excellent regeneration characteristics and is widely used in the treatment of estrogen chemicals in municipal wastewater. 

**Figure 8:**
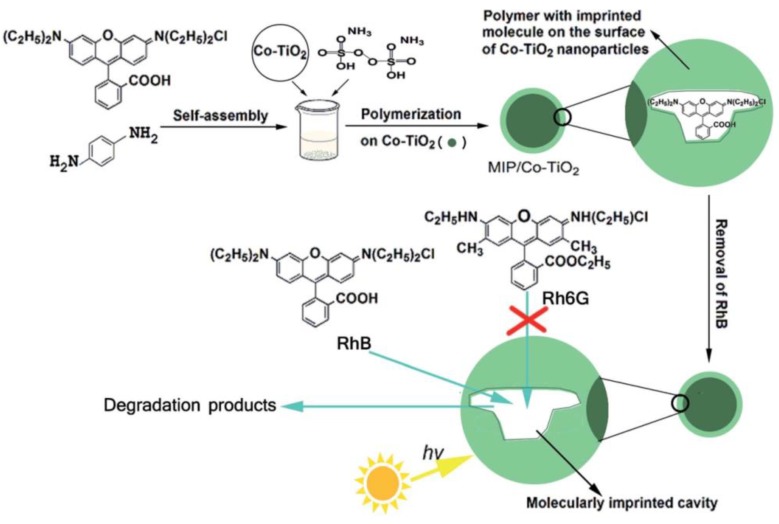
The schematic route for preparation of MIPs/Co–TiO_2_ nanocomposites with RhB as the template molecule and its use in photocatalytic degradation. Reprinted with permission from ref [87]. Copyright 2016 American Chemical Society.

Rhodamine B (RhB) is a kind of typical synthetic cationic dye, which has strong fluorescence in solution. RhB can cause the light transmittance of the water body to drop and destroy the ecological environment even at low concentrations. He et al. [86] used RhB as the template molecule and successfully prepared MIPs_RhB_–PPy/TiO_2_ (polyacrylamide/titania) by surface molecular imprinting using RhB as the template molecule. Static and dynamic binding experiments and selective experiments showed that MIPs_RhB_–PPy/TiO_2_ had strong affinity and adsorption capacity, fast adsorption rate and good recognition selectivity to RhB. Selective photocatalytic degradation experiments showed that the apparent rate constant of RhB to photodegradation of MIPs_RhB_–PPy/TiO_2_ was 0.0158 min^−1^, 3.6 times higher than that of NIP-PPy/TiO_2_ (0.0044 min^−1^). The MIPs_RhB_–PPy/TiO_2_ surface was introduced into the imprinting cavity, which had higher photocatalytic selectivity for RhB than NIP-PPy/TiO_2_ in visible light. In addition, MIPs_RhB_-PPy/TiO_2_ had higher reusability and stability. Furthermore, Liu et al. [87] used RhB as the template molecule, OPDA as the functional monomer and APS as the initiator to prepare imprinted polymer-coated Co-doped TiO_2_ (MIPs/Co-TiO_2_) nanocomposites by surface molecular imprinting technology (Figure 8). Compared with non-imprinted Co-doped TiO_2_ nanocomposites (NIPs/Co-TiO_2_), The k value for the photodegradation of RhB over MIPs/Co–TiO_2_ nanocomposites was 0.03606 min^−1^, being 337.3% of RhB over Co–TiO_2_ nanoparticles (0.01069 min^−1^) and 215.7% of RhB over NIP/Co–TiO_2_ nanocomposites (0.01672 min^−1^), which proved that MIPs/Co-TiO_2_ nanocomposites showed higher photodegradation and selectivity to RhB. In addition, MIPs/Co-TiO_2_ nanocomposites exhibited high stability.

Perfluorinated chemicals (PFCs) have high chemical stability, long-lasting fouling and bioaccumulation potential [95], posing a potential risk to human health and aquatic organisms [96]. Some PFCs, especially perfluorooctanoic acid (PFOA) and perfluorooctanesulfonate (PFOS), are often detected in surface water, sediments and WWTPs. Wu et al. [88] prepared the molecularly imprinted polymer modified TiO_2_ nanotubes (MIP-TiO_2_NTs) by using PFOA as the template molecule, acrylamide as the functional monomer, azobisisobutyronitrile (AIBN) as the initiator, EGDMA as the crosslinking agent. They also tested the selective removal of PFOP in water and the result showed that the amount of PFOA adsorbed by MIP-TiO_2_ NTs was as high as 0.8125 μg/cm^2^. Compared with TiO_2_-NTs and NIP-TiO_2_NTs, MIP-TiO_2_ NTs not only have higher PFOA degradation rate but also improve the selectivity of target chemical substances significantly. So this study can provide a solution to handle fluorinated chemicals in life.

Norfloxacin is a synthetic antibacterial, mainly for anti-bacterial and anti-inflammatory. The global demand and use of such antimicrobial agents are particularly large, resulting in a large number of residues in the environment. Li et al. [89] used surface molecular imprinting technique to modify TiO_2_ particles (P25). The results of orthogonal experiment showed that the adsorption rate constant, maximum adsorption capacity and Langmuir constant of norfloxacin in MIPs were 0.49 g mg^−1^ min^−1^, 2.99 mg g^−1^ and 2.4 L mg^−1^, respectively. MIPs adsorbed norfloxacin more strongly than P25. In addition, the removal efficiencies of norfloxacin, ciprofloxacin, carbamazepine and phenol by MIPs were 76.99, 78.81, 7.88 and 2.68%, respectively, indicating that MIPs have a higher level of selectively for norfloxacin and fluoroquinolone with similar structures. At the same time, MIPs showed good photocatalytic performance and had stable removal efficiency for norfloxacin after 5 adsorption-regeneration cycle tests. It can be widely used to remove norfloxacin in aquatic environment.

Diclofenac (DIC) is still one of the most frequently detected pharmaceuticals in the water environment and it has been detected in both the influents and effluents of wastewater treatment plants at concentrations up to mg/L level. Cícero Coelho et al. [91] prepared a molecularly imprinted photocatalyst containing a low loading of TiO_2_ and Cu_2_O-doped TiO_2_ by using a precipitation polymerization method, which showed target-specific molecular binding and degradation for DIC. In contrast to non-target reference molecules, the MIPs and the composite photocatalysts exhibited superior specific target recognition for selective degradation of DIC. The degradation of DIC with MIP25 reached 62.5% after 300 min UV light irradiation, which is much higher than that achieved with NIP25 and with NIP.

In addition to estrogen chemicals, rhodamine B, perfluorides, norfloxacin and other common pollutants; some other waste organic matters have also been handled by MIPs modified TiO_2_ nanomaterials. Wu et al. [83] successfully prepared N-F co-doped molecularly imprinted TiO_2_ (MIP-NFTs) by ethanol-hydrothermal method using 2-nitrophenol (2-NP) and 4-NP as the template molecule and n-butyl titanate as the crosslinking agent. The k value for the photodegradation of 2-NP over 2-NP/MIP-NFTs was 0.05233 min^−1^, being 267% of that over NIP-NFTs (0.01962 min^−1^) and the k value of 4-NP over 4-NP/MIP-NFTs was 0.03734 min^−1^, being 198% of that over NIP-NFTs (0.01882 min^−1^). Compared with NIP-NFTs, MIP-NFTs showed higher photocatalytic activity and selectivity of target pollutants under simulated sunlight. In addition, the reuse of MIP-NFTs showed a high degree of stability and reusability for its inorganic structure. Deng et al. [97] synthesized mesoporous molecularly imprinted nanosized TiO_2_ with molecular recognition and photocatalytic ability by using CTAB and urea as the structure-directed agent. Taking 4-nitrophenol as the target pollutant, they found that the adsorption capacity of 4-nitrophenol was about 3 times higher than that of non-imprinted TiO_2_ (control TiO_2_) and the relative selectivity coefficient was 3.645. In addition, the mesoporous enzyme molecularly imprinted TiO_2_ had good photocatalytic activity on 4-nitrophenol under simulated sunlight. The experimental results showed that the molecular imprinting technique and the fusion of mesoporous structures are the powerful bases for constructing highly efficient photocatalysts with high selectivity for certain organic pollutants. Shen et al. [90] used diethyl phthalate (DEP) as the template molecule to synthesize the inorganic molecularly imprinted polymers (IMIPs) photocatalyst to degrade DEP. The apparent rate constant k for the photodecomposition of DEP was 0.12 min^−1^ over IMIPs-P25, being 14.0, 9.2, 4.6 and 2.5 times that over TiO_2_/SiO_2_ (0.013 min^−1^), NIP-P25 (0.018 min^−1^) and P25 (0.049 min^−1^), which indicated that the IMIPs-P25 has stronger photocatalytic activity than other materials. It had been found experimentally that the IMIPs layer provided the molecular recognition capability for the photocatalyst and can achieve the selective adsorption and rapid mineralization of target pollutants with low concentration in other high concentration non-target pollutants. Compared to pure TiO_2_ photocatalyst (Degussa P25), IMIPs-coated TiO_2_ photocatalyst nearly eliminated the production of toxic aromatic by-products. In addition, the new photocatalyst consisted entirely of inorganic compounds, resistant to photochemical attack and had long life in the photocatalytic process. Shen et al. [84] prepared modified surface molecularly imprinted TiO_2_ by using OPDA as a template molecule, MAA as a functional monomer. The k value of the target 4-NP over 4NP-P25 is 0.045 min^−1^, being 346% and 188% of that over NIPs-P25 (0.013 min^−1^) and P25 (0.024 min^−1^) and the k value of 2-NP over 2NP-P25 is 0.040 min^−1^, being 333% of that over NIP-P25 (0.012 min^−1^) and 160% of that over P25 (0.025 min^−1^). Photocatalytic degradation experiments confirmed that the molecular recognition provided by the MIPs layer for photocatalysts can result in the selective photocatalytic degradation of the target pollutants, that is, the selective removal of low concentration and high toxicity organic pollutants from the contaminated water. In addition, Sharabi et al. [98] took diisopropylmethylphosphonate (DIMP) and diethylhydroxymethylphosphonate (DEHMP) as the template molecule, TiOSO_4_ as the titanium source and functional monomer to prepare molecularly imprinted polymer by a sol-gel method. It was found that the mineralization rate was improved by a factor of 3–4 in the presence of imprinted substrate and the substrate imprinted with DEHMP were very effective in the degradation of the homolog DIMP. This experiment proposed a method that a substrate with good affinity can be used to obtain a high surface concentration of active sites in the molecule and it is possible to avoid the aggregation problem that may occur when the target pollutant has low affinity with the substrate. To remove Rose Bengal (RB) dye from industrial wastewater selectively and quantitatively, Ahmed et al. [92] prepared a new MIP chitosan-TiO_2_ nanocomposite (CTNC). The prepared MIP nanoparticles exhibited a high surface area (95.38 m^2^/g) with relatively uniform mesoporous channels, which allowed an exceptional uptake of the dye (the maximum adsorption capacity: *Q*_m_ = 79.365 mg/g) and reflected the high selectivity of the prepared MIP compared to pure chitosan.

### 3.2. Applications of TiO_2_ Nanomaterials Based MIPs in Sensors

During the past decades, MIPs have been regarded as an attractive tool for the analysis of complex matrices because of their special specificity towards the target and high stability. They have been combined with several transducers for the development of different sensors. An overview of various applications of TiO_2_ nanomaterials based MIPs in sensors construction is listed in Table 3.

#### 3.2.1. Applications of TiO_2_ Nanomaterials Based MIPs in Electrochemical Sensors

**Figure 9:**
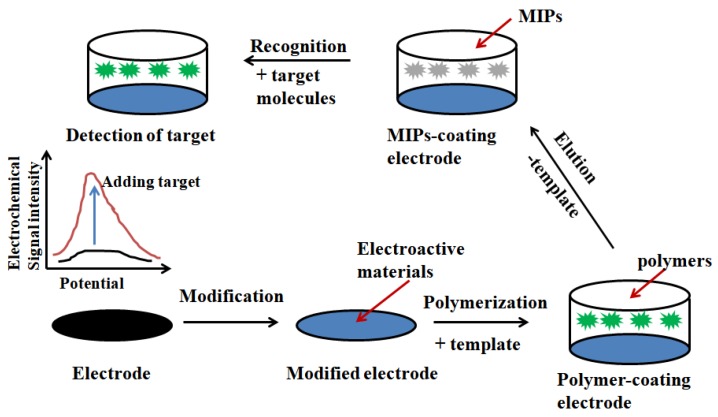
Schematic illustration of the fabrication and application of MIPs-based electrochemical biosensors. Adapted from ref [10].

Electrochemical sensor (Figure 9) is the use of target substances react with specific inductive elements to generate the detection signal and then this detection signal through a specific transducer can be converted to a target electrical signal proportional to the concentration of identifiable, so as to achieve the purpose of qualitative or quantitative detection and analysis of target substance [108,109]. The combination of molecular imprinting technology and electrochemical sensors can improve the selectivity and sensitivity of electrochemical sensors [110,111], shorten the reaction time and reduce the cost of instrumentation [112].

Bagheri et al. [99] took sol-gel method to synthesis Fe_3_O_4_ @ SiO_2_ @ TiO_2_-MIPs nanocomposites and used trimethylolpropane trimethacrylate as the crosslinking agent, 2,2′-azobis (2-methyl propionitrile) as the initiator. It was found that the introduction of Fe_3_O_4_ @ SiO_2_ @ TiO_2_ nanocomposites into MIPs enhanced the electrochemical signal and recognition ability of the sensor for the detection of ephedrine. This simple and selective sensor not only had good sensitivity to ephedrine but also had excellent reproducibility and stability. The detection range was 0.009–2.8 mM and the detection limit was 0.0036 mM. The sensor has been successfully used to detect ephedrine in biological fluids and drug samples, indicating that the sensor can be a useful tool in clinical and toxicology laboratories.

Wang et al. [57] used p-tert-butylcalix [6] arene and ethanol as functional monomers to prepare Phi-NO_2_ sensors based on molecularly imprinted TiO_2_ by liquid deposition. Due to the interaction between the molecularly imprinted binding site and the template, the deposited film showed better sensitivity, stability, selectivity and reproducibility to the analyte. The characterization of the imprinted TiO_2_ liquid-phase deposited films by X-ray diffraction (XRD) and electrochemical evidenced the feasibility of this method. The detection limit of Phi-NO_2_ was 0.04 μM and the detection range was 0.1–50 μM. This simple and efficient method has great potential for application to the construction of sensors. Wang et al. [58] used p-tert-butylcalix [6] arene as the functional monomer and acetaminophen(APAP) as the template to prepare an electrochemical sensor based on a molecularly imprinted TiO_2_ thin film. The sensor showed good sensitivity, selectivity and reproducibility for acetaminophen. The detection limit of acetaminophen was 0.2 μM and the detection range was 5.0–80.0 μM and 8.0–5.0 μM.

Qian [100] synthesized a recognition element of molecularly imprinted films (MIFs) on the surface of a Ti/TiO_2_ electrode for highly selective and sensitive electrochemical detection of bisphenol A (BPA). The blotting sites can selectively rebind BPA through hydrogen bonds, resulting in an increase in equilibrium currents in amperometric detection, which can electrochemically sense BPA. The detection limit of BPA was 1.3 nM and the detection range was 4.4–0.13 mM. Combined with the high selectivity of MIFs and the high sensitivity of electrochemistry, the MIFs based electrochemical sensor has showed high sensitivity and selectivity to BPA, with outstanding reusability, practicability and reliability.

#### 3.2.2. Applications of TiO_2_ Nanomaterials Based MIPs in Photoelectrochemical Sensors

Photochemistry (PEC) sensor is a device that detects the process with the conversion of light energy to chemical energy and electricity [113,114]. The principle is that the photoactive can react with the analyte under the light irradiations. According to the relationship between the charge of photocurrent or photovoltaic voltage with the concentration of analyte, the quantitative analysis of the analyte could be achieved [115]. Photoelectrochemical analysis based on this phenomenon has the characteristics of high sensitivity, simple equipment and easy miniaturization [116,117]. In addition, PEC uses light as an excitation signal and an electrical signal as a detection signal, which is contrary to the traditional electrochemical analysis process and shows a higher sensitivity and selectivity [118,119]. TiO_2_ is the most widely used metal oxide photoactive material. The combination between TiO_2_ and MIPs can greatly improve the photoelectrical response of TiO_2_ [120,121].

Thanhthuy et al. [101] fabricated a novel PEC sensor by imprinting a selective layer on highly ordered and vertically aligned nanotube arrays. The photocurrent was proportional to the concentration of PFOS in the range of 0.5–10 μM with a detection limit of 86 ng mL^−1^. The prepared sensor (MIP/TiO_2_ NATs) showed highly sensitive and selective characters to PFOS in water samples. Some other high concentration pollutants (such as twenty times 2,4-dichorophenoxyacetic acid (2,4-D) and two times PFOA) did not interfere the determination of PFOS. The selective determination of PFOS in pollution water can make the application of the PEC sensor become a reality.

**Figure 10:**
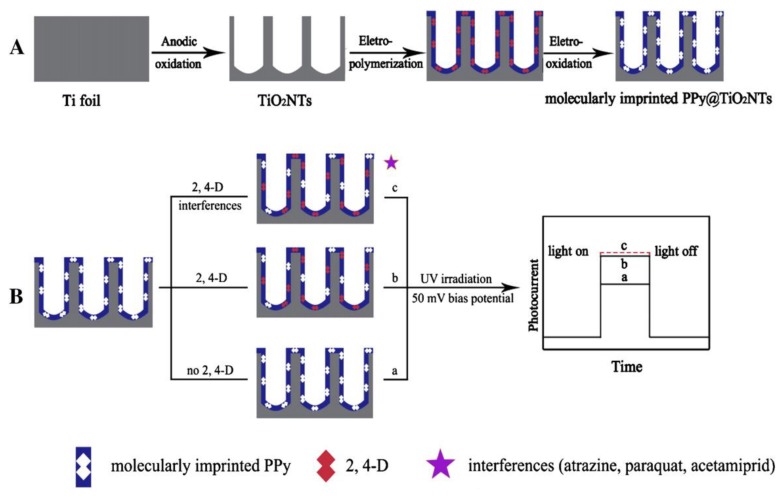
Schematic illustration for (**A**) fabrication and (**B**) detection mechanism of the photoelectrochemical sensor. Reprinted with permission from ref [102]. Copyright 2011 American Chemical Society.

Shi et al. [102] prepared a PEC sensor with a low detection limit based on modified TiO_2_ nanotubes (TiO_2_ NTs) to detect 2,4-D (2,4-D, a putative endocrine disruptor which lacks electrochemical activity) selectively and sensitively (Figure 10). The detection range was 0.5–13 μM and the detection limit was 10 nM. Thereafter, Lu et al. [103] fabricated a novel PEC sensor based on vertically aligned TiO_2_ nanobutes with surface molecularly imprinted PPy to detect another endocrine disruptor BPA. The photocurrent was proportional to the concentration of BPA in the range of 4.5–108 nM, with a detection limit of 2.0 nM. The results showed that the prepared sensor had highly selectivity and sensitivity and can determine BPA from other high concentration substances in water samples. What’s more, the PEC sensor showed good applicability and high stability in real water, which made a successful attempt in developing highly selective and sensitive PEC sensors for endocrine disruptors monitoring. 

Wang et al. [104] imprinted o-phenylenediamine (o-PD) monomers and chlorpyrifos(CPF) template molecules on gold nanoparticle-modified TiO_2_ nanotubes to prepare molecularly imprinted polymer films for the detection of CPF molecular by photoelectrochemical method. The experimental results showed that under visible light irradiation, the excited electrons were migrated from CPF to AuNPs and then to the conduction band of TiO_2_NTs. Under the optimal experimental conditions, the photocurrent was proportional to the concentration of CPF in the range of 0.05–10 μM with a detection limit of 0.96 nM. The MIPs-based PEC sensor was extremely specific and promising in applications of organochlorine pesticides and can be used to detect CPF in green vegetables. Sun et al. [105] also constructed a PEC sensor based on MIP modified hierarchical branched TiO_2_ nanorods (B-TiO_2_ NRs) by the hydrothermal method, which can detect CPF sensitively and efficiently. The PEC sensing platform is developed for the detection of CPF in the linear range from 0.01 to 100 ng·mL^−1^ with a low detection limit of 7.4 pg·mL^−^^1^. Later, Wang et al. [106] used lindane instead of CPF as a template molecule and aminothiophenol as a functional monomer to construct a molecularly imprinted polymer film for the detection of lindane via the photoelectrochemical method. Similarly, the MIPs-based PEC sensor with a linear range from 0.1–10 μM and detection limit of 0.03 μM had excellent specificity and could be successfully applied to the identification and detection of lindane in real samples. 

In order to rapidly and accurately detect microcystin (MC-LR, a strong liver tumor promoter), Liu et al. [107] took MC-LR as the template molecule to prepare molecularly imprinted TiO_2_ coated multi-walled carbon nanotubes (MI-TiO_2_ @ CNTs) by the sol-gel method. The MI-TiO_2_ @ CNT PEC sensor with a linear range from 1.0 pM–3.0 nM and detection limit of 0.4 pM exhibited higher photooxidation capability to MC-LR compared to conventional TiO_2_ and non-imprinted (NI-) TiO_2_ @ CNTs. What’s more, the sensor had high photocurrent sensitivity and excellent selectivity. It can provide a promising PEC analysis platform for future generations.

### 3.3. Miscellaneous Applications of MIPs Modified TiO_2_ Nanomaterials in Other Fields

In addition to their use as synthetic receptors in sensor platforms, there is also a recent trend to employ MIPs in various applications that go beyond analytical detection. Takahara et al. [40] deposited a complex of *β*-CD/BPA and Ti (O-Bu-n)_4_ alternately on the QCM and prepared the BPA imprinted TiO_2_ film by the sol-gel method on the gas phase surface. They confirmed the film formation and sensitivity of TiO_2_/(*β*-CD/BPA) films by QCM frequency measurements. In addition, the sensitivity of the imprinted TiO_2_/(*β*-CD/BPA) film was as low as 50 ppb to BPA. This method has the potential of detecting various organic compounds in liquids and gases.

Geng et al. [122] prepared a new surface molecularly imprinted polymer based on nano-TiO_2_by using propazine (Pro) as the template molecule, EGDMA and 2,2′-dimethacrylate acid as the crosslinking agent, methacrylic acid as the functional monomer, and isobutyronitrile as the initiator. The tests on all kinds of properties of this MIPs showed that it had good adsorption capacity and high recognition selectivity for promethazine. In the meantime, it also presented good cross-selectivity with 2-chloro-4,6-bis(ethylamino)-1,3,5-triazine(simazine,Sim) and 2-chloro-4-diethylamino-isopropylamino-1,3,5-triazine (Atrazine, Atr). In addition, new SMIPs based on nano-TiO_2_ were used as solid phase extraction (SPE) materials and three pesticide residues in water, soil, corn plants and grain samples were extracted, purified and determined by MIPs-SPE and high performance liquid chromatography (HPLC). The result substantiated that the MIPs enabled the high selectivity and enrichment of Pro, Atr and Sim from complex environmental media. This technology provided an analytical platform for quantitative analysis of traces of Pro, Sim and Atr residues in multi-environment media and food sources.

Khoddami et al. [123] used 3-(2-aminoethylamino) propyltrimethoxysilane (AAPTS) as the functional monomer, tetraethyl orthosilicate as the crosslinking agent and Co (II) as the template and then synthesized Co (II) ions magnetic molecularly imprinted polymer (Fe_3_O_4_ @ TiO_2_ @ SiO_2_-IIP) using a sol-gel method. The magnetic ion imprinted polymer which had been consumed can be refreshed by simply washing with HNO_3_ aqueous solution and the adsorption capacity did not have a significant drop after up to seven cycles of testing, indicating that the Fe_3_O_4_ @ TiO_2_ @ SiO_2_-IIP is stable and reusable. In addition, the preparation of the compound was relatively easy and the experimental data fit the pseudo-second-order kinetic model, which was in good agreement with the Langmuir adsorption isotherm. Based on the properties demonstrated in this study, Fe_3_O_4_ @ TiO_2_ @ SiO_2_-IIP was a candidate for the selective determination of Co (II) in biological and environmental samples.

## 4. Conclusions and Outlook

This up-to date review has clearly shown that MIPs modified TiO_2_ nanomaterial has attained much attention because they combined the good photo catalytic characters of TiO_2_ and the excellent selectivity of MIPs. MIPs modified TiO_2_ nanomaterials have been prepared in a controlled way by applying different technologies and surface chemistry. Because of the better recognition ability, higher selectivity and stronger adsorption capacity than non-imprinted ones towards analytes especially when the analytes were of low concentration or in a mixture, MIPs modified TiO_2_ nanomaterials have exhibited remarkable advantages for the application in pollutant removal, sensors, separation and so forth. Many successful examples for the development of MIPs modified TiO_2_ nanomaterials and their applications have been reported. 

In spite of the tremendous progress that has been made in the MIPs modified TiO_2_ nanomaterials, many challenges summarizing in Table 1 (e.g., limited usage of visible light, applications in biology) remain to be addressed. These existing problems restricted the working efficiency and advanced applications of MIPs modified TiO_2_ nanomaterials. The main challenges includes: (1) Extending light utilization to visible light range and reducing the recombination of electron-holes of TiO_2_. TiO_2_ doping or choosing suitable monomers for imprinting could improve the photo related characters of the nanocomposite; (2) Enhancing the binding sites and binding capacities between template and monomers. New synthetic techniques, such as controlled radical polymerizations (CRPs), could be introduced in the MIPs modified TiO_2_ nanomaterials preparation process which would lead to improved affinity. (3) Preventing the collapse or deformation of the imprinted cavities on MIPs during the application procedure. Balancing the good affinity and the stability of the imprinted cavities is one of the significant factors to be considered. Presumably, the higher porosity polymers contains more cavities are easier to collapse leading to the changes in binding properties. Efficient imprinting technology or elution method should be studied; (4) Exploring imprinting methods to broaden target molecules from small molecules to biological macromolecules, such as proteins and even to living cells. TiO_2_ has been regarded as a biocompatible material but their real applications in biological areas or clinical trials are very rare. The development of MIPs modified TiO_2_ nanomaterials with biocompatible properties is a challenge that can be expected to yield a new generation of sensors materials for biomedical applications. (5) Decreasing or eliminating cross-selectivity, that is the binding to molecular similar to the native template. Initial template interactions with functional monomers largely determine the recognition properties the matrix. Therefore, it is necessary to seek suitable monomers capable of forming better, more stable and strong interactions with the template. Computer aided design would help to seek for the suitable monomers.

In short, with the continuous development of computer aided design method, synthesis methods and detection technologies, the theoretical system of MIPs modified TiO_2_ nanomaterials will be becoming more perfect and widely used. Great prospects of these synthetic materials in the sensor, catalyst, electrode array and so forth, can be seen in the future.

## Figures and Tables

**Table 1:** Advantages and disadvantages of MIPs modified TiO_2_ nanomaterials.

Materials	Advantages	Disadvantages	Ref
TiO_2_/MIPs	high oxidation efficiency nontoxicity great adsorption and photocatalytic capacity towards specific pollutants high photostability chemical inertness environmentally friendly nature low cost mechanical, thermal and chemical stability high binding affinity easy way of preparation at a large scale structure-activity predictability specific recognition wide practicability	low utilization of visible-light rapid recombination of photogenerated electron/hole pairs the limited and heterogeneity of the binding sites cross-selectivity leakage of template limited application in biology	[3,23,24,25]

**Table 2:** Application of MIPs modified TiO_2_ nanomaterials for photocatalytic degradation.

Template/Degraded Target	Monomer/Support/Synthesis Method	Characterization Techniques	Light Source	Absorption Amount of Degradation Target on MIPs	Reaction Rate Constant (k/min^−1^)	Ref
OPDA/2-NP, 4-NP	MAA/P25/SMIT	UV–vis, HRTEM, FTIR	250 W Philips high-pressure mercury lamp	0.84, 0.61 mg/g	0.01073, 0.00706	[84]
2-NP, 4-NP	Ti(O-nBu)_4_/TiO_2_@WO_3_/Sol-gel	XRD, SEM, UV–vis	300 W xenon lamp	1.593, 0.139 mg/g	0.00373	[42]
AOII	Ti(O-nBu)_4_/Fe-TiO_2_/Sol-gel	FESEM, EDS, XRD, UV–vis, FTIR	500 W mercury lamp	9.35 mg/g	0.5861	[45]
9-AnCOOH	Ti(O-nBu)_4_/TiO_2_ NTs/Sol-gel	XRD, DRS, SEM,	500W xenon arc lamp	0.22 mg/g	0.1046	[46]
TC	TiO_2_/LPD	ESEM, XRD	UV light irradiation	0.065 mg/g	0.00363	[59]
estrone	Fe_3_O_4_@SiO_2_@TiO_2_/LPD	TEM, FTIR, XRD	20 W UV light	2.62 mg/g	0.069	[62]
17β-estradiol	MAA/TiO_2_ NTs/precipitation polymerization	SPE, UV–vis, FTIR, XRD	8W mercury UV lamp	10 ng/L–1000 mg/L	0.0732	[85]
RhB	TiO_2_/SMIT	XRD, TEM, UV–vis	500 W Xenon lamp	3.40mg/g	0.0158	[86]
RhB	OPDA/Co-TiO_2_/SMIT	XRD, FTIR, XPS, SEM, TEM, UV–vis DRS	400 W metal halide lamp	0.48 mg/g	0.03606	[87]
PFOP	AA/TiO_2_ NTs/SMIT	XRD, FESEM, HPLC	23 W UV-C light lamp	0.812 𝜇g/cm^2^	0.0036	[88]
Norfloxacin	TiO_2_/SMIT	UV–vis	300W UV lamp	2.99 mg/g	0.0632	[89]
2-NP, 4-NP	Ti(OBu)_4_/Ethanol TiO_2_/hydrothermal method	XRD, SEM, UV–vis DRS, XPS	400 W metal halide lamp	1.33, 0.80 mg/g	0.05233, 0.03028	[83]
DEP	Al^3+^ doped TiO_2_@ SiO_2_/Sol-gel	XRD, TEM, FTIR	200W UV lamp	18.5 mg/g	0.12	[90]
DIC	MAA/CuP25/precipitation polymerization	XRD, SEM, TEM	UV light irradiation	8.6 mg/g	-	[91]
RB	Ti(OH)_4_/CTNC/Sol-gel	SEM, XRD, FTIR	UV light irradiation	79.356 mg/g	0.0702	[92]
2,4-DNP	OPDA/TiO_2_/SMIT	FESEM, FTIR, XRD, UV-vis DRS	300 W xenon lamp	7.16 mg/g	0.0026	[93]

**Table 3:** Applications of MIPs modified TiO_2_ nanomaterials for sensors construction.

Target (Analyze)	Monomer/Support/Synthesis Route	Techniques Used for Characterization	Detection Technique	Detection Range	LOD	Ref
ephedrine	MMA/Fe_3_O_4_@SiO_2_@TiO_2_/Sol-gel	FT-IR, XRD, SEM, TEM	EC	0.0090–2.8 mM	0.0036 mM	[99]
Phi-NO_2_	p-tert-butylcalix[6]arene ethanol/TiO_2_/LPD	XRD	EC	0.1–50 mM	0.04 μM	[57]
APAP	p-tert-butylcalix[6]arene ethanol/TiO_2_/LPD	AFM, UV–vis	EC	5–80 μM, 0.8–5 μM	0.2 μM.	[58]
BPA	p(AN-co-AA)/Ti-TiO_2_/SMIT	SEM, UV–vis, EDX	EC	4.4–0.13 mM	1.3 nM	[100]
PFOS	Acrylamide/TiO_2_ NTs/UV polymerization	FTIR, FESEM	PEC	0.5–10 μM	86 ng/mL	[101]
2,4-D	pyrrole/TiO_2_ NTs/electropolymerization	UV–vis DRS	PEC	0.5–13 μM	10 nM	[102]
BPA	Pyrrole/TiO_2_ NTs/electropolymerization	UV–vis, XRD, SEM	PEC	4.5–108 nM	2.0 nM	[103]
CPF	PoPD/TiO_2_NTs/electropolymerization	UV–vis, SEM	PEC	0.05–10 mM	0.96 nM	[104]
CPF	TiO_2_NRs/hydrothermal method	SEM, TEM	PEC	0.029–2.85 nM	0.021 pM	[105]
lindane	PoPD/TiO_2_ NTs/electropolymerization	UV-vis, SEM	PEC	0.1–10 μM	0.03 μM	[106]
MC-LR	MWCNTs/Sol-gel	DRS, XRD, XPS, TEM, UV-vis	PEC	1.0 pm–3.0 nM	0.4 pM	[107]

## List of Abbreviations

TiO_2_	titanium dioxide
MIPs	molecularly imprinted polymers
NIPs	non- imprinted polymers
MMIPs	magnetic-molecular imprinted polymers
CNTs	carbon nanotubes
SMIT	surface molecular imprinting technique
MAA	methacrylic acid
BSM	bensulfuron-methyl
KH570	3-(trimethoxysilyl) propylmethacrylate
Cys	cysteine
Cys@ZnS:TiO_2_ NPs	cysteine derivative modified TiO_2_ doped ZnS nanoparticle
AA	acrylamide
CMC	carboxymethyl cellulose
PVA	polyvinyl alcohol
APS	ammonium persulfate
2,4-D	2,4-dichlorophenoxyacetic acid
NBD	nitrobenzoxadiazole
TCPO	bis(2,4,6-trichlorophenyl)oxalate
APTS	3-aminopropyltriethoxysilane
GFM	grafting-from
DBT	dibenzothiophene
4-VP	4-vinylpridine
EGDMA	ethylene glycol dimethacrylate
β-CD	β-cyclodextrin
BPA	bisphenol A
QCM	quartz crystal microbalance
GPTMS	glycidoxy propyltrimethoxysilane
MIPs/Fe–TiO_2_	molecularly imprinted inorganic-framework Fe–TiO_2_ composites
AOII	acid orange II
4-NP	4-nitrophenol
2-NP	2-nitrophenol
LPD	liquid deposition method
TC	tetracycline hydrochloride
GA	L-glutamic acid
EDMA	ethylene glycol dimethacrylate
OPDA	ortho-phenylenediamine
4-CP	4-chlorophenol
2-CP	2-chlorophenol
EEs	Environmental Estrogens
RhB	Rhodamine B
PDA	phenylenediamine
PFCs	perfluorinated chemicals
PFOA	perfluorooctanoic acid
PFOS	perfluorooctane sulfonate
CTNC	chitosan-TiO_2_ nanocomposite
RB	Rose Bengal
AIBN	azobisisobutyronitrile
P25	a kind of TiO_2_ particles
CTAB	cetrimonium bromide
DEP	diethyl phthalate
IMIPs	inorganic molecularly imprinted polymers
DIMP	diisopropyl methylphosphonate
DEHMP	diethylhydroxymethylphosphonate
XRD	X-ray diffraction
Phi-NO_2_	O,O-dimethyl-(2,4-dichlorophenoxyacetoxyl)(30-nitrobenyl)methinephosphonate
APAP	acetaminophen
MIFs	molecularly imprinted films
BPA	bisphenol A
PEC	photochemistry
o-PD	o-phenylenediamine
MC-LR	microcystin
Pro	propazine
Sim	simazine
Atr	Atrazine
HPLC	high performance liquid chromatography
SPE	solid phase extraction
AAPTS	3-(2-aminoethylamino) propyltrimethoxysilane
